# Network Hubs in the Brain Have the Biggest Impact on Behavior

**DOI:** 10.1371/journal.pbio.1002178

**Published:** 2015-06-30

**Authors:** Janelle Weaver

**Affiliations:** Freelance Science Writer, Carbondale, Colorado, United States of America

## Abstract

Are memory deficits better explained by damage to individual brain areas or by an interruption in the flow of information among widely distributed networks in the prefrontal cortex? A new study highlights the importance of task-related function over anatomy. Read the Research Article.

The most highly evolved brain region in mammals is the prefrontal cortex, which regulates our thoughts, actions, and emotions through extensive connections with other brain regions. Studies in humans have shown that multiple parts of the prefrontal cortex are activated during memory tasks, but patients with damage to some of these areas do not always have memory problems. As a result, researchers have disputed whether memory deficits are caused by damage to individual brain areas subserving specific cognitive functions or by an interruption in the flow of information among widely distributed areas in the prefrontal cortex.

A recently proposed hypothesis reconciles these views by suggesting that cortical areas form a highly ordered network containing hubs that play a critical role in information processing, such that damage to a hub results in severe cognitive impairment. However, most investigations of network structure have relied on either anatomical studies or functional neuroimaging of spontaneous activity at rest, ignoring brain activity related to specific cognitive tasks.

In a study published this week in *PLOS Biology*, Yasushi Miyashita of the University of Tokyo School of Medicine and his colleagues used functional magnetic resonance imaging (fMRI) and a novel simulated-lesion method in monkeys to show that virtual damage to a prefrontal cortex hub, which was the most highly interconnected with other brain areas activated during a memory task, was predicted to produce the most severe memory impairment. By contrast, virtual damage to a highly interconnected prefrontal cortex hub that was previously identified in anatomical tracer studies was not predicted to produce severe memory problems. According to the authors, these findings lay the foundation for precisely predicting the behavioral and cognitive impact of injuries or surgical interventions in the human brain.

In the new study, the researchers conducted fMRI in two macaque monkeys performing a temporal-order judgment memory task (Fig 1). The monkeys first viewed a sequence of pictures of objects, and then they saw two of these pictures simultaneously presented side by side on the screen. Using a joystick, the monkeys indicated which of the two objects was presented more recently. The fMRI results showed significant activity in multiple brain areas in prefrontal cortex, including areas 9/46d and 8Ad. The patterns of connections between brain areas during the memory task revealed that area 9/46d was the most highly interconnected area (a “functional hub”) in the prefrontal cortex, whereas area 8Ad was not a significant hub in the network. By contrast, previous anatomical tracer studies, which do not take into account changes in the connections between brain areas due to the task at hand, indicated that area 8Ad, but not area 9/46d, is a central anatomical hub in the prefrontal cortex of the macaque brain.

**Fig 1:**
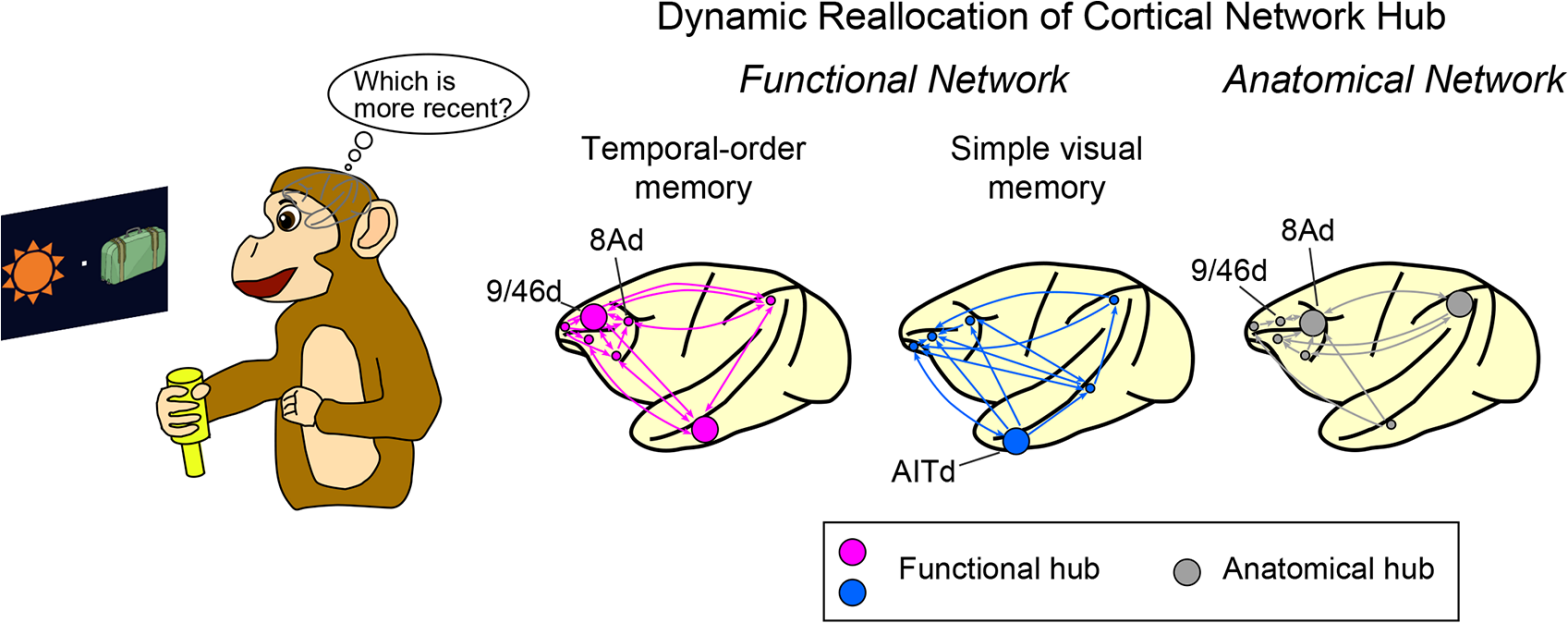
The location of a functional hub changes depending on cognitive demands. Lesions to the functional, rather than the anatomical, hub disrupt memory. *Image credit*: *Takahiro Osada*.

To test if the location of the functional hub changes depending on task demands, the researchers conducted additional fMRI experiments in the same monkeys using a simple memory task. A picture of an object was shown, and after a delay, this object was simultaneously presented side by side with another object. The monkeys then chose which of the two objects they previously saw. In contrast to the more difficult temporal-order memory task as well as anatomical tracer studies, the simple context-free memory task revealed a central functional hub in the anterior inferior temporal region. Taken together, the findings indicate that distinct network hubs in the brain support different cognitive tasks and that functional hubs related to brain activity do not correspond to anatomical hubs defined by patterns of neuronal connections.

To estimate the impact of virtual brain damage, the researchers used algorithms to predict the degree to which removal of each area would reduce accuracy in the temporal-order judgment memory task. Based on the fMRI results, area 9/46d was the only area in prefrontal cortex predicted to impair memory performance when removed from the network. Surprisingly, removal of brain areas that were more highly interconnected during the task was predicted to have a greater impact on memory performance. These findings are supported by a past study showing that damage to area 9/46d, but not area 8Ad, impairs performance on this task. By contrast, the interconnectedness of brain areas, as defined by anatomical connections, was not predicted to influence the severity of behavioral impairment.

By providing evidence that functional network hubs in the brain play a critical role in information processing and behavioral performance, the study explains why brain damage in the prefrontal cortex does not always produce memory deficits in patients. On a practical note, the findings could be used to predict the severity of impairment following brain surgery or injuries. In the end, this information could guide clinical interventions to spare damage to critical network hubs, as well as optimize rehabilitation strategies to improve the recovery of behavioral and cognitive abilities, resulting in better clinical outcomes for patients.
